# Phenolic Compounds from Araticum Pulp (*Annona crassiflora* Mart.) Modulate Inflammatory Targets: Insights from *in Vitro* and *in Silico* Approaches

**DOI:** 10.1021/acsomega.5c12027

**Published:** 2026-05-19

**Authors:** Amanda Cristina Andrade, Henrique Silvano Arruda, Lívia Mateus Reguengo, Ana Sofia Martelli Chaib Saliba, Severino Matias de Alencar, Glaucia Maria Pastore

**Affiliations:** † Department of Food Science and Nutrition (DECAN), School of Food Engineering (FEA), University of Campinas (UNICAMP), Campinas, São Paulo 13083-862, Brazil; ‡ Department of Agri-Food Industry, Food and Nutrition, Luiz de Queiroz College of Agriculture (ESALQ), University of São Paulo (USP), Piracicaba, São Paulo 13418-900, Brazil

## Abstract

Araticum (*Annona crassiflora* Mart.), a native fruit from the Brazilian Cerrado, is known for its high content of phenolic compounds, particularly flavonoids. These biomolecules exhibit diverse biological activities, including potential anti-inflammatory effects. In this context, the present study aimed to evaluate the effect of the modulation of inflammatory targets by araticum pulp through an integrative approach combining *in vitro* assays and *in silico* analyses. UHPLC-Q-Orbitrap-MS/MS identified 75 compounds, predominantly flavonoids and phenolic acids. HPLC-DAD analysis evidenced high levels of flavonoids (mainly epicatechin and procyanidin B2), but low carotenoid content. Araticum pulp demonstrated a strong antioxidant capacity, particularly through the scavenging of hydroxyland peroxyl radicals, and exhibited consistent activity in ABTS^•+^, DPPH, and FRAP assays. Moreover, araticum significantly inhibited NF-κB activation by 60.98% in LPS-stimulated RAW 264.7 macrophages. Molecular docking shows stable interactions of major flavonoids from araticum with inflammatory targets, especially IL1R/IL1β, JAK1, and JAK2. These results provide novel insights into the bioactive profile of araticum pulp, evidencing the fruit as a natural source of compounds with antioxidant activity and modulatory effects on inflammatory signaling and cytokine production.

## Graphical abstract

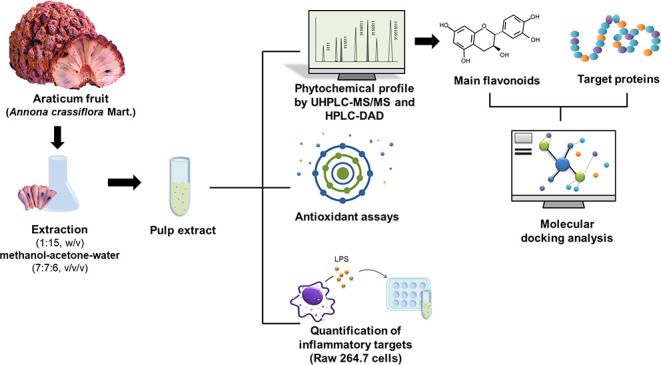

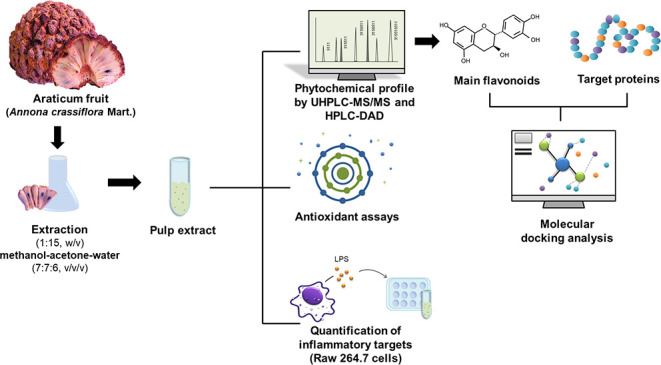

## 1 Introduction

Oxidative stress arises from a persistent imbalance between reactive oxygen species (ROS) and antioxidant compounds, producing excess free radicals. This condition can result from excessive ROS generation or failures in the body’s defense systems, leading to disrupted redox homeostasis and damage to biomolecules such as lipids, proteins, and nucleic acids, as well as to tissues.^1,2^ These cellular damages can trigger an inflammatory response, which in turn further contributes to oxidative stress, creating a continuous cycle between inflammatory mechanisms and oxidative stress.^3^

Inflammation is a fundamental defense mechanism to control injury or infection through cellular and molecular interactions. Following tissue injury, a chemical signaling cascade is initiated, causing the chemotaxis of phagocytic leukocytes. These immune cells infiltrate the damaged tissue andproduce cytokines and ROS, amplifying inflammatory responses.^4,5^ ROS can promote inflammation through the upregulation of genes that encode pro-inflammatory cytokines, contributing to cytotoxicity and/or by the production of nuclear transcription factor-κB (NF-κB), a transcription factor that induces the expression of inflammatory mediators, e.g., interleukin (IL)-1β, IL-6, IL-8, tumor necrosis factor (TNF)-α, inducible nitric oxide synthase (iNOS), and cyclooxygenase-2 (COX-2).^6,7^ Although acute inflammation is essential, its dysregulation can lead to chronic inflammation and pathogenesis of several diseases, including cardiovascular diseases, inflammatory bowel diseases, diabetes, arthritis, and cancer.^2,5^

In this context, phenolic compounds, plant-derived secondary metabolites, exhibit strong antioxidant capacity, attributable to their ability to sequester or inhibit reactive species, chelate metal ions, eliminate free radicals, activate antioxidant enzymes, inhibit oxidative enzymes, and alter signaling transduction pathways. These mechanisms occur independently or in specific combinations, thereby helping to reduce oxidative stress.^8,9^ Due to these properties, plant phenolic compounds have attracted increasing scientific interest for their remarkable antioxidant and anti-inflammatory potential, which makes them promising agents for the prevention and management of chronic non-communicable diseases.^6,7^

Brazil is recognized for its rich diversity of native fruits, distributed across its vast territory, which encompasses six biomes (Amazon, Cerrado, Atlantic Forest, Caatinga, Pantanal, and Pampa). These biomes provide specific climatic conditions that favor the cultivation of fruits with unique sensory characteristics and notable nutritional and pharmacological properties, making them valuable for food and medicinal applications.^10,11^ However, many native fruit species are relatively unknown and remain underexploited, limited primarily to family farming and local markets, where they are consumed either fresh or processed into products such as jelly, jam, pulp, juices, and ice cream.^12^

Among these fruits, *Annona crassiflora* Mart., commonly known as araticum, is a native fruit to the Brazilian Cerrado that stands out for its high nutritional value and bioactive compounds content, including phenolic compounds, annonaceous acetogenins, carotenoids, and alkaloids.^13,14^ These compounds, especially phenolics, have been associated with several biological activities, such as antioxidant, anti-inflammatory, anti-Alzheimer, anticancer, and antibacterial effects.^15^

Despite the recognized bioactive properties of araticum pulp, its anti-inflammatory potential remains insufficiently explored, particularly in cellular models. Moreover, there is a lack of studies regarding the molecular mechanisms involved in its anti-inflammatory effects, as well as limited evidence from computational approaches, such as molecular docking. Molecular docking is a computational tool widely used to predict the binding affinity and interactions of bioactive compounds with target proteins,^16^ to support and elucidate these interactions.

Considering the growing interest in the bioactivity of phenolic compounds and the injurious effects of oxidative stress and inflammation on human health, this study aimed to characterize the phytochemical profile and antioxidant capacity of araticum pulp and to evaluate its effect on the modulation of inflammatory targets using RAW 264.7 macrophages as a cellular model. Furthermore, molecular docking analyses were conducted to investigate potential interactions between the main phenolic compounds from araticum pulp and inflammation-related target proteins, offering new insights into their possible mechanisms of action and providing a theoretical basis for the future application of araticum pulp as a functional food.

## 2 Materials and Methods

### 2.1 Plant Material and Sample Preparation

Fully ripe araticum fruits were collected in Carmo do Paranaíba, Minas Gerais, Brazil (19°00′03″ south latitude, 46°18′58″ west longitude, and 1061 m altitude). A voucher specimen (UEC 197249) has been deposited in the Herbarium of the Institute of Biology at the University of Campinas, Brazil (Herbarium UEC). The Genetic Heritage Management Board (CGen) authorized accessing genetic heritage under the number AF88C29.

Freeze-dried araticum pulp was mixed with methanol-acetone–water (7:7:6, v/v/v) at a solid-to-liquid ratio of 1:15. The mixture underwent ultrasound-assisted extraction in an ultrasonic bath (UNIQUE, model UCS-2850, 25 kHz, 120 W, São Paulo, SP, Brazil) at room temperature for 30 min. After extraction, the samples were centrifuged at 4000×*g* for 5 min at 5 °C (Hettich Zentrifugen, model Rotanta 460R, Tuttlingen, Germany). The supernatant was collected and the *pellet* was re-extracted twice under the same conditions.^17^ The obtained supernatants were combined and the resulting extract was stored at −20 °C until analysis.

### 2.2 Determination of Total Phenolic Content (TPC)

Total phenolic content was determined according to Roesler et al.^18^ with modifications. The extract (30 μL), 10% (v/v) Folin-Ciocalteu reagent (150 μL), and 7.5% (w/v) sodium carbonate solution (120 μL) were mixed. The mixture was incubated for 6 min at 45 °C, and absorbance was read (SPECTROstar Nano, BMG Labtech, Ortenberg, Germany) at 760 nm against a blank. The result was expressed as milligrams of gallic acid equivalents per gram of dried pulp (mg GAE/g dw).

### 2.3 Determination of Total Flavonoid Content (TFC)

The total flavonoid content was determined by mixing 30 μL of the extract with 110 μL of ultrapure water and 8 μL of 5% (w/v) NaNO_2_, followed by standing for 5 min. Subsequently, 8 μL of 10% (w/v) AlCl_3_ was added, and after 6 min, 50 μL of 1 M NaOH solution and 70 μL of water were added. The absorbance was read in a microplate reader (SPECTROstar Nano, BMG Labtech, Ortenberg, Germany) at 510 nm against a blank.^19^ The result was expressed as milligrams of catechin equivalents per gram of dried pulp (mg CE/g dw).

### 2.4 Determination of Condensed Tannins Content (CTC)

The condensed tannins content analysis was conducted using the method of Arruda, Pereira, de Morais et al.^17^ with modifications. Briefly, 20 μL of the extract were mixed with 180 μL of 4% (w/v) vanillin in methanol and 90 μL concentrated HCl. The mixture was incubated for 20 min at room temperature, and the absorbance was measured at 500 nm using a microplate reader (SPECTROstar Nano, BMG Labtech, Ortenberg, Germany). The result was expressed as milligrams of catechin equivalents per gram of dried pulp (mg CE/g dw).

### 2.5 Determination of Carotenoids by HPLC-DAD

The carotenoids of freeze-dried araticum pulp (1 g) were exhaustively extracted with acetone, followed by liquid–liquid partition to petroleum ether/diethyl ether (1:1, v/v) by washing with distilled water. After the liquid–liquid partition, the residual water was removed from the organic portion using anhydrous sodium sulfate, and the organic solvents were evaporated from the extract under vacuum (temperature <38 °C) until dryness. The dried carotenoid extract was solubilized in methyl *tert*-butyl ether (MTBE) and filtered before HPLC injection, according to the procedures described by Chisté and Mercadante.^20^

Chromatographic separation of carotenoids was conducted using a reverse-phase YMC-C30 column (250 × 4.6 mm id, 5 μm particle size, YMC Europe, Schermbeck, Germany) into a high-performance liquid chromatography system equipped with a diode array detector (Agilent Technologies 1260 Infinity II, Waldbronn, Germany). The column oven temperature was maintained at 29 °C and the flow rate was 0.9 mL/min. The mobile phases A (methanol) and B (MTBE) were applied following the elution gradient: 0–30 min (5–30% B) and 30–50 min (50% B). The UV–vis spectra were recorded between 200 and 600 nm, and the chromatograms were processed at 450 nm. The carotenoids were tentatively identified based on the following combined information: elution order on the C30 column, UV–vis [λ_max_], spectral fine structure (%III/II), *cis*-peak intensity (%A_B_/A_II_), coelution with available authentic standards, and comparison with the literature.^20,21^ The result was expressed as micrograms per gram of dried pulp (μg/g dw).

### 2.6 Determination of Phytochemical Profile by UHPLC-Q-Orbitrap-MS/MS

The phytochemical profile of the araticum pulp extract was analyzed using a Thermo Ultimate 3000 chromatographic system (Waltham, MA, USA) coupled to a Q-Exactive mass spectrometer equipped with an electrospray ionization (ESI) source, operated in negative electrospray ionization (ESI^–^) mode. Chromatographic separation was performed on a Poroshell 120 SB-Aq column (100 × 2.1 mm i.d., 2.7 μm particle size, Agilent Technologies, Santa Clara, CA, USA), maintained at 40 °C, with a gradient elution at a flow rate of 0.45 mL/min. The mobile phase was comprised of 0.1% formic acid in water (eluent A) and acetonitrile containing 0.1% formic acid (eluent B) with a gradient elution program of 0–1 min (5% B); 1–10 min (5–18% B); 10–13 min (18–70% B); 13–15 min (70–100% B); 15–17 min (100% B); 17–19 min (100–5% B); and 19–22 min (5% B).^22^

The mass spectrometer (MS) operated in ESI^–^ mode, with the following parameters: desolvation gas flow at 51 L/min, auxiliary gas flow at 13 L/min, sweep gas flow at 3 L/min, spray voltage at 2.5 kV, capillary temperature at 266 °C, RF lens S at 50, and auxiliary gas temperature at 431 °C. The instrument scanned a mass range of 100–1500 Da at a resolution of 70,000 (AGC target of 3 × 10^6^ and a maximum injection time (IT) of 100 ms). For MS/MS, a resolution of 17,500 was used (AGC target of 1 × 10^5^ and a maximum IT of 50 ms). The top five most intense precursor ions were fragmented using stepped normalized collision energies (NCE) of 25, 30, and 35 eV, with an isolation window of 3.0 *m*/*z*. Xcalibur software (version 4.3) was used for data acquisition and qualitative analysis. Nontargeted phytochemical profiling was based on exact mass (limit of 10 ppm), fragmentation patterns, comparison with literature data, and phytochemical databases such as MassBank (http://massbank.jp), METLIN Metabolite (https://metlin.scripps.edu), and HMDB (https://hmdb.ca).

### 2.7 Determination of Phenolic Compounds by HPLC-DAD

The Dionex UltiMate 3000 system, coupled to a diode array detector (Thermo Fisher Scientific, Waltham, MA, USA), was used to quantify phenolic compounds from araticum pulp, according to the method developed and validated by Borsoi et al.^9^ Chromatographic separation was conducted using a reverse-phase AcclaimTM 120 A C18 column (250 × 4.6 mm i.d., 5 μm particle size, Thermo Fisher Scientific, Waltham, MA, USA). The column oven temperature was maintained at 32 °C. The flow rate was 0.5 mL/min with an injection volume of 20 μL.

The mobile phases consisted of 0.1% formic acid in deionized water (eluent A) and acetonitrile (eluent B), following the elution gradient: 0–5 min (5% B); 5–27 min (5–29% B); 27–33 min (35% B); 33–45 min (35–50% B); 45–50 min (95% B); and 50–60 min (5% B). UV–vis absorption spectra of the standards and samples were measured at a wavelength between 190 and 800 nm. Phenolic compounds were identified by comparing UV–vis spectra and retention times to authentic standards, and quantified using different wavelengths of 260, 280, 320, or 360 nm, depending on the compound. The result was expressed as micrograms per gram of dried pulp (μg/g dw).

### 2.8 Antioxidant Capacity Assays

#### 2.8.1 DPPH Assay

The DPPH assay was adapted from the method by Arruda, Pereira, de Morais et al.^17^ For analysis, an aliquot of 50 μL of extract was combined with 250 μL of DPPH in ethanol (0.004%, w/v). The mixture was incubated for 30 min at room temperature. After incubation, the absorbance was measured at 517 nm against a blank in a microplate reader (SPECTROstar Nano, BMG Labtech, Ortenberg, Germany). The result was expressed as micromoles of Trolox equivalents per gram of dried pulp (μmol TE/g dw).

#### 2.8.2 ABTS^•+^ Assay

The ABTS^•+^ assay was determined using the method outlined by Re et al.^23^ with modifications. ABTS^•+^ radical was generated by mixing ABTS (7 mM) with potassium persulfate (140 mM) and incubating overnight. For the analysis, the ABTS solution was diluted in water until the absorbance of 0.70 ± 0.02 at 734 nm. Then, 50 μL of extract were mixed with 250 μL of ABTS^•+^ solution. After 6 min, absorbance was measured at 734 nm, using a microplate reader (SPECTROstar Nano, BMG Labtech, Ortenberg, Germany). The result was expressed as micromoles of Trolox equivalents per gram of dried pulp (μmol TE/g dw).

#### 2.8.3 Ferric Reducing Antioxidant Power (FRAP) Assay

The FRAP assay was performed as described by Benzie and Strain^24^ with modifications. FRAP reagent was prepared in a 10:1:1 (v/v/v) ratio of acetate buffer (0.3 mol/L, pH 3.6), 2,4,6-tris­(2-pyridyl)-s-triazine (TPTZ; 10 mmol/L), and ferric chloride (20 mmol/L). For the reaction system, 20 μL of extract, 180 μL of FRAP solution, and 60 μL of deionized water were added. Posteriorly, it was incubated for 30 min at 37 °C, and the absorbance was measured at 595 nm using a microplate reader (SPECTROstar Nano, BMG Labtech, Ortenberg, Germany). The result was expressed as micromoles Trolox equivalents per gram of dried pulp (μmol TE/g dw).

#### 2.8.4 Oxygen Radical Absorbance Capacity (ORAC) Assay

In the total ORAC assay (T-ORAC), randomly methylated β-cyclodextrin (RMCD, 7% w/v in acetone: water, 1:1 v/v) was used as solvent. The reaction was carried out with 20 μL of extract, 120 μL of fluorescein (0.387 μg/mL), and 120 μL of 2,2′-azobis­(2-methylamidinopropane)-dihydrochloride (AAPH; 108 mg/mL). For the hydrophilic ORAC assay (H-ORAC), the potassium phosphate buffer (pH 7.4, 75 mM) was used as solvent. The reaction consisted of 20 μL of extract, 120 μL of fluorescein (0.387 μg/mL), and 60 μL of AAPH (108 mg/mL). Fluorescence intensity was recorded every minute for 80 cycles at 37 °C with excitation and emission wavelengths of 485 and 520 nm, respectively, using a microplate reader (NOVOstar, BMG Labtech, Offenburg, Germany).^25^ The result was expressed as micromoles of Trolox equivalents per gram of dried pulp (μmol TE/g dw).

#### 2.8.5 Hydroxyl Radical (^•^OH) Scavenging Activity

The hydroxyl radical scavenging assay was performed as described by Mariutti et al.^26^ with modifications. The reaction contained 50 μL of carbonate buffer (0.5 M, pH 10), 50 μL of extract, 50 μL of luminol solution (100 μM) in the carbonate buffer (0.5 M, pH 10), 50 μL of FeCl_2_-EDTA solution (125 and 500 μM), and 50 μL of H_2_O_2_ solution (17.5 mM). After 5 min of incubation, luminescence was read at 37 °C on a microplate reader (Molecular Devices, LLC, Sunnyvale, CA, USA). The result was expressed as IC_50_ (μg/mL dw).

#### 2.8.6 Hypochlorous Acid (HOCl) Scavenging Activity

The HOCl was made using NaOCl solution (1%, pH 6.2) and adjusted with H_2_SO_4_ solution (10%). The HOCl solution concentration was adjusted by dilution in phosphate buffer (100 mM, pH 7.4) and reading absorbance at 235 nm, until obtaining a 5 μM HOCl solution (molar absorption coefficient 100 M^–1^cm^–1^). The dihydrorhodamine 123 (DHR) was prepared in the phosphate buffer at 1.25 μM. For the reaction system, 100 μL of extract, 100 μL of phosphate buffer, 50 μL of DHR, and 50 μL of HOCl were mixed. Fluorescence (excitation: 485 nm, emission: 528 nm) was read at 37 °C using a microplate reader (Molecular Devices, LLC, Sunnyvale, CA, USA).^27^ The result was expressed as IC_50_ (μg/mL dw).

#### 2.8.7 Superoxide Radical (•O_2_^–^) Scavenging Activity

The superoxide radical scavenging activity was performed by a reaction with 100 μL of nicotinamide adenine dinucleotide hydrate (NADH, 166 μM), 50 μL of nitroblue tetrazolium (NBT, 107.5 μM), 50 μL of phenazine methosulfate (PMS, 2.7 μM), and the extract (100 μL) in potassium phosphate buffer (19 mM, pH 7.4). After 5 min of incubation in the dark, absorbance was read at 560 nm on a microplate reader (Molecular Devices, LLC, Sunnyvale, CA, USA).^27^ The result was expressed as IC_50_ (μg/mL dw).

### 2.9 Cellular Assessment of Inflammatory Targets

The assays were performed according to the method described by Saliba et al.^28^ using murine macrophages Raw 264.7 transfected with the gene NF-κB Luciferase (BPS Bioscience). The Raw 264.7 were cultured in Dulbecco’s Modified Eagle’s Medium (DMEM) supplemented with 10% (v/v) of heat-inactivated fetal bovine serum (FBS), 1% (v/v) of penicillin/streptomycin sulfate, and 1% (v/v) of glutamine, and incubated at 37 °C and 5% CO_2_. After reaching about 80% confluence, the cells were scratched and used in subsequent analysis.

#### 2.9.1 Cell Viability

The cells were cultured at 6 × 10^4^ cells/well in a 96-well plate and incubated for 24h at 37 °C, 5% CO_2_, and controlled humidity. After incubation, the medium was discarded, and the cells were treated with different concentrations of araticum pulp extract (50, 100, 500, and 1000 μg/mL), while the control was treated only with DMEM medium (M). The plate was incubated for 4h at 37 °C and 5% CO_2_. Then, the treatment was removed, 200 μL of 3-(4,5-dimethylthiazol-2-yl)-2,5-diphenyltetrazolium bromide (MTT; 0.05 mg/mL) solution were pipetted, and the plate was reincubated for 3h. Afterward, the MTT solution was discarded, 100 μL of dimethyl sulfoxide (DMSO) were added, and absorbance was measured at 570 nm on the microplate reader (SpectraMax M3, Molecular Devices, LLC, Sunnyvale, CA, USA). The cell viability was quantified as a percentage relative to the control, which was considered to have 100% of viability.

#### 2.9.2 NF-κB Activation and Levels of TNF-α and CXCL2/MIP-2

Raw 264.7 cells (3 × 10^5^ cells/well) were seeded in 24-well plates and incubated for 12h at 37 °C in a humidified atmosphere with 5% CO_2_. After incubation, the DMEM was removed, and the cells were pretreated with araticum pulp extract at concentrations of 50, 100, and 500 μg/mL diluted in DMEM, for 30 min at 37 °C, 5% CO_2_ in a humidified atmosphere. Thereafter, the cells were stimulated with 100 ng/mL of lipopolysaccharide (LPS) and reincubated for 4h under the same conditions described.

The positive control (LPS) consisted of LPS-activated cells without pretreatment, whereas the negative control (M) consisted of cells maintained in DMEM only. After 4 h, the supernatant was recovered and used to quantify TNF-α and CXCL2/MIP-2 (R&D Systems, Minneapolis, MN, USA) using the enzyme-linked immunosorbent assay (ELISA), following the manufacturer’s instructions. The results were expressed in pg/mL.

To evaluate NF-κB activation, 50 μL of lysis buffer (TNT) were added to Raw 264.7 cells, and 10 μL of lysed cells were mixed with 25 μL of luciferase reagent (luciferin at 0.5 mg/mL) in a 96-well plate. The luminescence was immediately measured using a microplate reader (SpectraMax M3, Molecular Devices, LLC, Sunnyvale, CA, USA). The result was expressed in the percentage of luminescence relative units.

### 2.10 Molecular Docking Analysis

Molecular Docking Analysis was performed as described by Borsoi et al.^9^ Software programs used in this study were Open Babel, PyMOL version 1.5.03 Open Source, and AutoDock. The 3D structures of catechin, epicatechin, procyanidin B2, and rutin were retrieved from the PubChem database (http://pubchem.ncbi.nlm.nih.gov/). PubChem CID, molecular weight, and chemical structures of the mentioned ligands are available in the Supporting Information (Table S1). The optimized structures were used as initial conformations for molecular docking studies.

The crystal structures of IL-6 (PDB ID: 1ALU), NF-κB (PDB ID: 3GUT), STAT3 (PDB ID: 4E68), TNF-α (PDB ID: 2AZ5), IL-1β (PDB ID: 2NVH), JAK1 (PDB ID: 6BBU), JAK2 (PDB ID: 3FUP), JAK3 (PDB ID: 6AAK), IL-23 (PDB ID: 5NDJ), IL-6R/IL-6 (PDB ID: 1P9M), IL-17A (PDB ID: 8DYF), IL-1R/IL-1β (PDB ID: 1ITB), and MIP-2 (PDB ID: 3N52) were retrieved from the Protein Data Bank (http://www.rcsb.org/). All water molecules, ligands, heteroatoms, and nonessential chains were removed from the protein structures. Polar hydrogen atoms were added, AD4 atom type was assigned, missing atoms were repaired, and Kollman charges were assigned and distributed throughout the residue. Finally, all target proteins were subjected to energy minimization before proceeding with the docking studies.

Active site prediction was developed on the online server PrankWeb (https://prankweb.cz/). The binding sites considered for each protein were those involved in binding along the NF-κB, JAK-STAT, IL-23/IL-17, and TNF-α pathways, as reported in the literature. Grid box coordinates were calculated based on X, Y, and Z coordinates of each amino acid included in the binding site (Table S2). The grid box was positioned around the center of the binding site with grid point spacing (0.375 Å), dimensioned enough to fit all protein residues that could be involved in the docking process, and AutoGrid was used to calculate the grid maps.

Docking simulations were performed using AutoDock v4.2, employing rigid target proteins and flexible ligands. The simulations were carried out using the Lamarckian Genetic Algorithm (LGA), an integrated algorithm in AutoDock, with an increased population size of 300 to enhance search efficiency. In AutoDock, each possible orientation of the ligand within the binding site is treated as an ’individual’ in the genetic algorithm. Each docking run was repeated 50 times, generating 50 docked conformations per ligand. AutoDock software estimates binding energy, inhibition constant, intermolecular energy, internal energy, and torsional energy, based on free energy functions. The binding energy was used to represent the rank of the docking poses.

### 2.11 Statistical Analysis

All analyses were independently performed in three replicates. Data are presented as the mean ± standard deviation (SD). The data from the inflammatory targets were assessed by one-way ANOVA and Tukey’s post hoc test. The statistical analyses were conducted at a 5% significance level (*p* < 0.05) using Minitab software version 18.1 (Minitab Inc., State College, PA, USA), and GraphPad Prism software (version 8.0.2) was used to plot the graphs.

## 3 Results and Discussion

### 3.1 Total Phenolic Content (TPC), Total Flavonoid Content (TFC), and Condensed Tannin Content (CTC)

The Folin-Ciocalteau method for TPC determination is the most commonly used in laboratories due to its speed, low cost, simplicity, and minimal equipment and reagent requirements.^29^ As shown in Table 1, the TPC from araticum pulp was 23.75 mg GAE/g dw, corresponding to 6.18 mg GAE/g fresh weight (fw). According to the classification proposed by Vasco et al.^30^ fruits are categorized into low (<1 mg GAE/g fw), medium (1–5 mg GAE/g fw), and high (>5 mg GAE/g fw) levels of TPC. From this classification, araticum pulp may be regarded as a fruit with a high content of phenolic compounds.

**Table 1:** Total Phenolic Content, Total Flavonoid Content, and Condensed Tannin Content in the Araticum Pulp^a^

**Parameters**	**Content**
Total phenolic content (mg GAE/g dw)	23.75 ± 0.21
Total flavonoid content (mg CE/g dw)	35.29 ± 2.07
Condensed tannins content (mg CE/g dw)	34.42 ± 0.90

a CE: catechin equivalents; dw: dry weight; GAE: gallic acid equivalent.

Arruda et al.^10^ and Arruda, Pereira, and Pastore^25^ reported similar TPC values in araticum pulp, with 21.74 and 26.20 mg GAE/g dw, respectively. In contrast, considerably higher TPC levels were observed in other studies, such as those by Stufassa et al.^31^ and Arruda et al.,^32^ with 45.58 and 46.70 mg GAE/g dw, respectively. These variations in TPC values among studies may be due to extraction methods, particle size of the sample, sample-to-solvent ratio, pH, and the type and polarity of the extraction solvent.^33^ Other possible causes include edaphoclimatic conditions, cultivation practices, harvest timing, and storage conditions, which influence the stability, concentration, and phytochemical composition of plants.^34^

Araticum pulp showed high and comparable content of TFC and CTC, corresponding to 35.29 and 34.42 mg CE/g dw, respectively (Table 1). In the same way, Arruda, Pereira, de Morais et al.^17^ reported a comparable TFC and CTC values (13.51 and 13.67 mg CE/g dw, respectively) in araticum pulp. Condensed tannins (proanthocyanidins) are formed by the condensation of flavan-3-ol units, such as catechin and epicatechin,^35^ supporting that tannins contribute substantially to the TFC value and explain the close similarity between these parameters.

Indeed, CTC accounts for around 97% of the TFC, a result corroborated by the individual phenolic profile obtained byHPLC-DAD analysis (See Table 4 in Section 3.4. In particular, 1163.52 μg/g of the total 1200.07 μg/g of identified flavonoids (∼97%) correspond to flavan-3-ols (catechin, epicatechin, and procyanidins). Previous studies also identified monomers (catechin and epicatechin) and oligomers (procyanidins) of flavan-3-ol as the main flavonoids in araticum pulp.^10,17^

**Table 2:** Chromatographic Profile, UV–Visible Characteristics, and Contents of Carotenoids from the Araticum Pulp Obtained by HPLC-DAD

**Carotenoid** ^a^	**Content (μg/g dw)** ^b^	**r.t. (min)** ^c^	**λ**_**max**_ **(nm)** ^d^	**%III/II**	**%A**_**B**_ **/A**_**II**_
epoxy-carotenoid^e^	0.57 ± 0.05	7.6	399, 421, 448	69	0
not identified	0.48 ± 0.05	9.8	400, 426, 450	n.c.	n.c.
lutein^e^	1.59 ± 0.29	13.7	420, 444, 470	59	0
zeaxanthin^f^	0.94 ± 0.06	16.1	420, 450, 477	23	0
α-carotene^g^	<LOQ	29.5	420, 445, 470	30	0
β-carotene^h^	2.29 ± 0.20	33.6	420, 450, 477	18	0
**total carotenoids**	5.87 ± 0.26				

a Tentative identification based on UV–visible, relative HPLC retention times, and published data.

b Mean ± standard deviation (*n* = 3).

c Retention time on YMC-C30 column (250 × 4.6 mm id, 5 μm particle size, YMC Europe, Schermbeck, Germany).

d Characteristic maximum absorption wavelengths corresponding to the peaks and shoulders in the UV–vis absorption spectra of carotenoids in a linear gradient of methanol/MTBE.

e The peaks were quantified as equivalent of lutein.

f The peaks were quantified as equivalent of zeaxanthin.

g The peaks were quantified as equivalent of α-carotene.

h The peaks were quantified as equivalent of β-carotene. <LOQ = below the limit of quantification (0.41 μg/mL). n.c.: not calculated.

Flavonoids can act as antioxidant and anti-inflammatory agents, mediating macrophages and controlling pro-inflammatory molecules. Furthermore, tannins are important bioactive compounds, recognized for their astringent properties, as well as anti-inflammatory, antimutagenic, wound healing, antioxidant, and antibacterial activities.^36^ This information evidence the potential contribution of these compounds to the biological properties of araticum pulp, providing beneficial effects on health and well-being.

### 3.2 Carotenoids Determination by HPLC-DAD

Carotenoids are a diverse group of natural, fat-soluble pigments, responsible for the red, orange, and yellow coloration in many fruits and vegetables.^37^ In the present study, six carotenoids were detected in araticum pulp by HPLC-DAD, and five of them were tentatively identified (Table 2).

The carotenoids are generally classified into carotenes (e.g., α-carotene, β-carotene, and lycopene) and xanthophylls (e.g., lutein, zeaxanthin, cryptoxanthin, and astaxanthin). Structurally, carotenes are hydrocarbons, while xanthophylls are oxygenated derivatives of carotenes.^38^ β-carotene (2.29 μg/g dw) and lutein (1.59 μg/g dw) were the major carotenoids identified in araticum pulp, together representing 66.10% of the total carotenoid content. According to the classification proposed by Britton and Khachik,^39^ which categorizes the content of each carotenoid as low (0–1 μg/g), moderate (1–5 μg/g), high (5–20 μg/g), or very high (>20 μg/g), araticum pulp can be considered a moderate source of β-carotene and lutein, although it showed low levels of other carotenoids such as epoxy-carotenoid, zeaxanthin, and α-carotene.

The higher content of β-carotene in araticum pulp is expected, as it is the most common carotenoid found in plant-based foods. β-carotene is a provitamin A compound, essential for maintaining vision, supporting immune function, and promoting cell growth. Meanwhile, lutein is produced from the hydroxylation of α-carotene and can be accumulated in the human retina, promoting benefits for eye health. In addition, carotenoids demonstrate important biological activities, including antioxidant, anti-inflammatory, photoprotective, anticarcinogenic, and cardioprotective effects.^37,38^

### 3.3 Phytochemical Profile by UHPLC-Q-Orbitrap-MS/MS

Table 3 presents the retention time (min), tentative identification for each phytochemical found in the araticum pulp, molecular formula, error (ppm), and main MS/MS fragment ions with the exact masses of precursor ions (negative ionization mode). A total of seventy-five compounds of different classes were tentatively annotated and characterized in the araticum pulp based on MS and MS/MS data, including nine organic acids and derivatives, thirty-one phenolic acids, and thirty-five flavonoids.

**Table 3:** Identified or Tentatively Annotated Phytochemicals in the Araticum Pulp by UHPLC-Q-Orbitrap-MS/MS under Negative Ion Mode^a^

**ID**	**r.t. (min)**	**Identified/Tentatively Annotated Compound**	**Molecular Formula**	**Observed** ***m/z*** **value**	**Theoretical** ***m/z*** **value**	**Error (ppm)**	**Characteristic MS/MS Fragments**
*Organic acids and derivatives*							
1	0.66	Hydroxyadipic acid	C_6_H_10_O_5_	161.0458	161.0450	4.97	101.0247, 99.0451
2	0.73	2-Furoic acid	C_5_H_4_O_3_	111.0087	111.0082	4.50	111.0085, 67.0088
3	0.74	Malic acid	C_4_H_6_O_5_	133.0143	133.0137	4.51	115.0039, 71.0135
4	0.83	Citric acid	C_6_H_8_O_7_	191.0200	191.0192	4.19	129.0200, 111.0087, 87.0089
5	0.85	Succinic acid	C_4_H_6_O_4_	117.0193	117.0188	4.27	99.0086, 73.0296
6	1.05	Shikimic acid	C_7_H_10_O_5_	173.0457	173.0450	4.05	173.0464, 155.0351, 131.0827, 129.0560, 111.0087
7	1.47	Ascorbic acid	C_6_H_8_O_6_	175.0253	175.0243	5.71	115.0403, 87.2409, 71.0135
8	1.61	n-Propylmalic acid	C_7_H_12_O_5_	175.0614	175.0606	4.57	131.0826, 115.0403, 113.0618, 85.0661
9	4.39	Tuberonic acid hexoside	C_18_H_28_O_9_	387.1672	387.1655	4.39	207.1038, 163.1133
*Phenolic acids and derivatives*							
10	1.31	Leonuriside A isomer 1	C_14_H_20_O_9_	331.1044	331.1029	4.53	168.0429, 153.0200
11	1.37	Salicylic acid isomer 1	C_7_H_6_O_3_	137.0246	137.0239	5.11	93.0349
12	1.40	Protocatechuic acid glucoside	C_13_H_16_O_9_	315.0731	315.0716	4.76	153.0566, 152.0118, 123.0458, 109.0299, 108.0219
13	1.53	Hydroxytyrosol hexosylpentoside isomer 1	C_19_H_28_O_12_	447.1528	447.1503	5.59	153.0555, 123.0454
14	1.61	Hydroxybenzoic acid hexoside	C_13_H_16_O_8_	299.0781	299.0767	4.68	137.0248
15	1.68	Leonuriside A isomer 2	C_14_H_20_O_9_	331.1043	331.1029	4.23	168.0433, 153.0191
16	1.81	Caffeoylsucrose isomer 1	C_21_H_28_O_14_	503.1413	503.1401	2.39	341.0892, 179.0358, 161.0251, 135.0461
17	1.88	Hydroxytyrosol hexosylpentoside isomer 2	C_19_H_28_O_12_	447.1527	447.1503	5.37	153.0554, 123.0453
18	1.99	Vanillic acid hexoside isomer 1	C_14_H_18_O_9_	329.0887	329.0873	4.25	167.0358, 152.0118, 123.0451, 108.0220
19	2.08	Protocatechuic acid pentoside isomer 1	C_12_H_14_O_8_	285.0623	285.0610	4.56	153.0201, 152.0126, 123.0315, 109.0305, 108.0215
20	2.17	Salicylic acid isomer 2	C_7_H_6_O_3_	137.0246	137.0239	5.11	93.0349
21	2.19	Caffeic acid hexoside isomer 1	C_15_H_18_O_9_	341.0883	341.0873	2.93	179.0360, 161.0249, 135.0455
22	2.22	Caffeoylsucrose isomer 2	C_21_H_28_O_14_	503.1412	503.1401	2.19	179.0351, 161.0248
23	2.30	Syringic acid hexoside isomer 1	C_15_H_20_O_10_	359.0995	359.0978	4.73	197.0469, 182.0222, 153.0552, 138.0324
24	2.33	Caffeic acid hexoside isomer 2	C_15_H_18_O_9_	341.0884	341.0873	3.22	179.0359, 161.0254, 135.0453
25	2.40	Dihydrocoumaroyl hexoside	C_15_H_20_O_8_	327.1088	327.1080	2.45	165.0563, 147.0452
26	2.65	Syringic acid hexoside isomer 2	C_15_H_20_O_10_	359.0995	359.0978	4.73	197.0468, 182.0222, 153.0571, 138.0324
27	2.94	Vanillic acid hexoside isomer 2	C_14_H_18_O_9_	329.0887	329.0873	4.25	167.0355, 123.0445
28	3.04	Protocatechuic acid pentoside isomer 2	C_12_H_14_O_8_	285.0624	285.0610	4.91	153.0202, 152.0114, 109.0304, 108.0220
29	3.18	p-Coumaric acid glucoside	C_15_H_18_O_8_	325.0938	325.0923	4.61	163.0410, 145.0302, 119.0508
30	3.23	Caffeic acid hexoside isomer 3	C_15_H_18_O_9_	341.0890	341.0873	4.98	179.0367, 161.0242, 135.0460
31	3.51	Caffeic acid	C_9_H_8_O_4_	179.0351	179.0344	3.91	179.0363, 135.0450, 134.0373, 107.0499
32	3.70	Vanillic acid hexoside isomer 3	C_14_H_18_O_9_	329.0889	329.0873	4.86	167.0355, 123.0445
33	3.87	Chlorogenic acid	C_16_H_18_O_9_	353.0887	353.0873	3.97	191.0565, 161.0248, 135.0462, 134.0382
34	4.11	Ferulic acid glucoside	C_16_H_20_O_9_	355.1040	355.1029	3.10	193.0515, 175.0411, 134.0382
35	6.06	Caffeoylshikimic acid	C_16_H_16_O_8_	335.0784	335.0770	4.18	179.0362, 161.0258, 135.0458
36	7.78	Caffeoyltyramine isomer 1	C_17_H_17_NO_4_	298.1088	298.1079	3.02	178.0519, 135.0460
37	9.57	Pinoresinol hexoside	C_26_H_32_O_11_	519.1882	519.1866	3.08	357.1382, 151.0408, 136.0170
38	9.58	Pinoresinol	C_20_H_22_O_6_	357.1351	357.1338	3.64	151.0399, 136.0171
39	10.28	Caffeoyltyramine isomer 2	C_17_H_17_NO_4_	298.1091	298.1079	4.03	178.0531, 135.0450
40	10.61	Syringaresinol glucoside	C_28_H_36_O_13_	579.2086	579.2078	1.38	417.1615, 181.0518
*Flavonoids and derivatives*							
41	3.14	Procyanidin B dimer isomer 1	C_30_H_26_O_12_	577.1379	577.1346	5.72	407.0789, 289.0712, 161.0245, 125.0253, 109.4116
42	3.18	Catechin	C_15_H_14_O_6_	289.0732	289.0712	6.92	245.0830, 221.0840, 151.0403, 137.0255, 125.0252, 109.0300
43	3.63	Procyanidin B trimer isomer 1	C_45_H_38_O_18_	865.2008	865.1980	3.24	407.0786, 289.0754, 287.0583, 161.0241, 125.0249
44	3.88	Procyanidin B trimer isomer 2	C_45_H_38_O_18_	865.2054	865.1980	8.55	407.0773, 289.0725, 287.0554, 161.0255, 125.0252
45	4.00	Procyanidin B dimer isomer 2	C_30_H_26_O_12_	577.1392	577.1346	7.97	407.0798, 289.0718, 161.0249, 125.0242, 109.0297
46	4.41	Epicatechin	C_15_H_14_O_6_	289.0730	289.0712	6.23	245.0855, 221.0849, 151.0403, 137.0256, 125.0245, 109.0299
47	4.52	Procyanidin B dimer isomer 3	C_30_H_26_O_12_	577.1375	577.1346	5.02	407.0756, 289.0741, 161.0240, 125.0250, 109.0292
48	4.87	Procyanidin B trimer isomer 3	C_45_H_38_O_18_	865.2051	865.1980	8.21	407.0798, 289.0714, 287.0589, 161.0244, 125.0251
49	5.30	Procyanidin A dimer isomer 1	C_30_H_24_O_12_	575.1225	575.1189	6.26	289.0719, 245.0847, 151.0394, 125.0251, 123.0445, 109.0303
50	5.69	Procyanidin B dimer isomer 4	C_30_H_26_O_12_	577.1382	577.1346	6.24	407.0759, 289.0742, 161.0240, 125.0250
51	6.14	Procyanidin A dimer isomer 2	C_30_H_24_O_12_	575.1232	575.1189	7.48	289.0713, 245.0843, 151.0411, 125.0251, 123.0455
52	6.40	Procyanidin B trimer isomer 4	C_45_H_38_O_18_	865.2048	865.1980	7.86	407.0802, 289.0715, 287.0591, 161.0244, 125.0251
53	6.51	Procyanidin A dimer isomer 3	C_30_H_24_O_12_	575.1235	575.1189	8.00	289.0722, 245.0837, 151.0407, 125.0248, 123.0455, 109.0291
54	6.59	Procyanidin B tetramer isomer 1	C_60_H_50_O_24_	1153.2679	1153.2614	5.64	407.0832, 289.0726, 287.0589, 161.0250, 125.0249
55	6.82	Procyanidin A dimer isomer 4	C_30_H_24_O_12_	575.1241	575.1189	9.04	289.0728, 245.0478, 151.0409, 125.0250
56	7.12	Procyanidin B tetramer isomer 2	C_60_H_50_O_24_	1153.2711	1153.2614	8.41	407.0774, 289.0725, 287.0554, 161.0250, 125.0249
57	7.14	Procyanidin B trimer isomer 5	C_45_H_38_O_18_	865.2009	865.1980	3.35	407.0790, 289.0735, 287.0598, 161.0249, 125.0242
58	7.16	Procyanidin A dimer isomer 5	C_30_H_24_O_12_	575.1218	575.1189	5.04	289.0739, 245.0829, 151.0408, 125.0250, 123.0454, 109.0292
59	7.91	Quercetin-3-*O*-pentosylhexoside	C_26_H_28_O_16_	595.1334	595.1299	5.88	301.0376, 300.0296
60	8.01	Procyanidin B dimer hexosylpentoside	C_40_H_40_O_22_	871.1899	871.1932	–3.79	407.0797, 289.0713, 287.0588, 161.0143, 125.0251
61	8.48	Procyanidin B dimer isomer 5	C_30_H_26_O_12_	577.1381	577.1346	6.06	407.0778, 289.0728, 161.0251, 125.0250
62	8.65	Quercetin-3-*O*-rutinoside (rutin)	C_27_H_30_O_16_	609.1484	609.1456	4.60	301.0385, 300.0304
63	8.88	Procyanidin B tetramer isomer 3	C_60_H_50_O_24_	1153.2681	1153.2614	5.81	407.0802, 289.0762, 287.0591, 161.0244, 125.0251
64	8.88	Kaempferol 3-*O*-xylosylglucoside	C_26_H_28_O_15_	579.1376	579.1350	4.49	285.0411, 284.0340
65	9.00	Procyanidin B trimer isomer 6	C_45_H_38_O_18_	865.2021	865.1980	4.74	407.0809, 289.0719, 287.0594, 161.0245, 125.0251
66	9.00	Quercetin-3-*O*-galactoside	C_21_H_20_O_12_	463.0884	463.0877	1.51	301.0347, 300.0267, 271.0258
67	9.04	Procyanidin B tetramer isomer 4	C_60_H_50_O_24_	1153.2644	1153.2614	2.60	407.0807, 289.0718, 287.0593, 161.0244, 125.0251
68	9.07	Procyanidin A dimer isomer 6	C_30_H_24_O_12_	575.1229	575.1189	6.96	289.0726, 245.0848, 151.0408, 125.0249, 123.0453, 109.0289
69	9.20	Phloretin-*C*-diglycoside	C_27_H_34_O_15_	597.1837	597.1820	2.85	387.1128, 257.1004
70	9.74	Kaempferol-3-*O*-rutinoside	C_27_H_30_O_15_	593.1526	593.1506	3.37	285.0404, 284.0344, 255.0320
71	9.88	Kaempferol-3-*O*-galactoside (trifolin)	C_21_H_20_O_11_	447.0948	447.0927	4.70	285.0401, 284.0352
72	10.07	Quercetin-3-*O*-arabinoside	C_20_H_18_O_11_	433.0782	433.0771	2.54	301.0338, 300.0206
73	10.18	Phlorizin	C_21_H_24_O_10_	435.1309	435.1291	4.14	273.0778, 167.0361
74	10.19	Kaempferol-3-*O*-glucoside (astragalin)	C_21_H_20_O_11_	447.0947	447.0927	4.47	285.0409, 284.0349, 255.0321
75	12.28	Luteolin	C_15_H_10_O_6_	285.0421	285.0399	7.72	285.0415, 151.0044, 133.0298

a r.t. (min): retention time; error (ppm): the difference between experimental mass and theoretical mass of the compound.

Organic acids not only influence the organoleptic properties of fruits but also demonstrate notable biological effects, including anti-inflammatory activity, modulation of immune function, anti-obesity effects, enhanced calcium absorption, and regulation of intestinal hormone secretion.^40^ Among the organic acids identified in the araticum pulp were malic, citric, succinic, shikimic, ascorbic acid, etc.

Malic and citric acids are the most common acids found in ripe fruits and act as important intermediates in metabolic pathways such as the Krebs cycle, playing an essential role in cellular energy transfer.^41,42^ In the study by Damiani et al.^43^ malic acid was the predominant in araticum pulp (958.5 μg/g fw), followed by citric acid (294 μg/g fw). Additionally, *n-*propylmalic acid, also detected in this study, is a derivative of malic acid formed during the fruit ripening.^10^

Ascorbic acid (vitamin C), another important organic acid identified, is a potent antioxidant capable of scavenging ROS. It also functions as a cofactor for several enzymes and plays a regulatory role in cell division, being considered an important compound in the prevention of cancer, cardiovascular diseases, and cataracts.^44^ Shikimic acid, on the other hand, is an intermediate compound in the biosynthetic pathway of aromatic amino acids, which serve as precursors for the synthesis of secondary metabolites, like phenolic compounds.^45^

Phenolic compounds are characterized by at least one aromatic ring with one or more hydroxyl groups. Hydroxyl and carboxyl groups in their structure enable phenolics to form conjugates with various molecules, including carbohydrates, organic acids, amines, and others.^8^ In this study, approximately 51% of the phenolic compounds tentatively identified in araticum pulp were found in their glycosylated form.

Glycosylation enhances the stability and solubility of phenolics and facilitates their storage in vacuoles. This mechanism protects the plants against the toxicity of their own phenolics and serves as a reserve for mobilization in response to biotic and abiotic stressors.^46^ These aspects may account for the predominance of glycosylated phenolic compounds observed in araticum pulp, suggesting that this structural form plays a key role in their stabilization and storage within the fruit tissues.

A total of thirty-one phenolic acids were tentatively identified in araticum pulp. Despite the wide diversity of these compounds, the most notable are caffeic acid and its derivatives (ten compounds), including caffeic acid, chlorogenic acid, two caffeoylsucrose isomers, three caffeic acid hexoside isomers, caffeoylshikimic acid, and two caffeoyltyramine isomers. Caffeic acid is one of the major representatives of hydroxycinnamic acids in the human diet and is well-known for its excellent antioxidant, anti-inflammatory, anticancer, and neuroprotective activity. These effects are largely related to its ability to downregulate interleukins (e.g., IL-6 and IL-1β) and NF-κB in the inflammatory response, and inhibit signaling pathways STAT3 and ERK1/2.^47,48^

The flavonoids were the main class identified in the araticum pulp, accounting for approximately 53% of the total phenolics (thirty-five compounds). Previous studies by Arruda et al.^10,49^ have also identified flavonoids as the primary class of phenolic compounds in araticum pulp, with catechin, epicatechin, rutin, dimers of procyanidin A, and dimers, trimers, and tetramers of procyanidin B being frequently reported.^10,33,49,50^ In particular, procyanidins were the most abundant compounds of the flavonoids, representing 63% of this class (twenty-two compounds). These compounds have antioxidant, anticancer, antimicrobial, anti-inflammatory, hypoglycemic, and antiallergic properties. In addition, their intake has been shown to change the gut microbiota and improve gut barrier function.^51^

Overall, the comprehensive phytochemical profiling of araticum pulp revealed a diverse composition mainly characterized by organic acids, phenolic acids, and flavonoids, with a high abundance of glycosylated phenolics, caffeic acid and its derivatives, and procyanidins. These findings not only corroborate previous reports on the phytochemical richness of araticum but also expand the understanding of its chemical composition, reinforcing its value as a promising source of health-promoting bioactive compounds.

### 3.4 Phenolic Compounds Content by HPLC-DAD

The individual phenolic content of araticum pulp by HPLC-DAD analysis is shown in Table 4. Thirty-three phenolic compounds were investigated, of which ten were identified and quantified, comprising five phenolic acids and five flavonoids. In the class of phenolic acids, the main compounds found were chlorogenic acid (20.26 μg/g dw), ferulic acid (8.24 μg/g dw), and caffeic acid (7.88 μg/g dw), while for flavonoids,epicatechin (711.14 μg/g dw), procyanidin B2 (439.20 μg/g dw), and rutin (28.19 μg/g dw) were the most predominant. In total, 1200.07 μg/g dw of flavonoids were quantified, representing 96.60% of the phenolic compounds.

**Table 4:** Phenolic Compounds Profile and Content in Araticum Pulp by HPLC-DAD^a^

**Class**	**Compound**	**Content (μg/g dw)**
Phenolic acids	4-Hydroxybenzoic acid	n.d.
	Benzoic acid	n.d.
	Caffeic acid	7.88 ± 0.24
	Chlorogenic acid (5-Caffeoylquinic acid)	20.26 ± 0.17
	Ferulic acid	8.24 ± 0.14
	Gallic acid	n.d.
	Gentisic acid (2,5-Dihydroxybenzoic acid)	n.d.
	*p*-Coumaric acid	3.47 ± 0.15
	Protocatechuic acid (3,4-Dihydroxybenzoic acid)	n.d.
	Sinapic acid	n.d.
	Syringic acid	n.d.
	*trans*-Cinnamic acid	2.33 ± 0.04
	Vanillic acid	n.d.
	α-Resorcylic acid (3,5-Dihydroxybenzoic acid)	n.d.
	**Total phenolic acids**	42.16 ± 0.10
Flavonoids	Apigenin	n.d.
	Apigetrin (Apigenin-7-*O*-glucoside)	n.d.
	Astragalin (Kaempferol-3-*O*-glucoside)	n.d.
	Catechin	13.14 ± 0.72
	Epicatechin	711.18 ± 5.21
	Hesperetin	t.r.
	Hyperoside (Quercetin-3-*O*-galactoside)	8.36 ± 0.13
	Kaempferol	n.d.
	Luteolin	n.d.
	Myricetin	n.d.
	Naringenin	n.d.
	Procyanidin A2	n.d.
	Procyanidin B1	n.d.
	Procyanidin B2	439.20 ± 4.29
	Quercetin	n.d.
	Quercitrin (Quercetin-3-*O*-rhamnoside)	n.d.
	Rutin (Quercetin-3-*O*-rutinoside)	28.19 ± 0.42
	Vitexin (Apigenin-8-*C*-glucoside)	n.d.
	Vitexin-2″-*O*-rhamnoside	n.d.
	**Total flavonoids**	1200.07 ± 6.93
	**Total phenolic compounds**	1242.24 ± 6.88

a dw: dry weight; n.d.: not detected; t.r.: traces.

These results, along with those in Tables 1 and 3, confirm that flavonoids are the predominant class of phenolic compounds in araticum pulp. Their high levels may be related to environmental conditions of the Brazilian Cerrado, including drought, high temperatures, intense UV radiation, and nutrient-poor soil.^35^ Under these stress conditions, plants can intensify secondary metabolism, leading to greater flavonoid accumulation to help protect against dryness, act as a UV filter, assist in heat acclimation, and defend against pathogens, since flavonoids play an essential role in plant defense and survival.^52^

Arruda, Pereira, de Morais et al.^17^ quantified six phenolic acids and four flavonoids in araticum pulp, with catechin and epicatechin (768.42 and 661.81 μg/g dw, respectively) as the predominant flavonoids, and caffeic and protocatechuic acids (124.31 and 97.92 μg/g dw, respectively) as the main phenolic acids. The total contents reached 1447.34 μg/g dw for flavonoid and 345.24 μg/g dw for phenolic acids. More recent studies by Felix Ávila et al.^53^ and Arruda et al.^49^ reported epicatechin and chlorogenic acid as the dominant phenolics in araticum pulp. Ramos et al.^33^ observed different phenolic profiles in araticum pulp from four distinct cities in Minas Gerais (Brazil), reinforcing that environmental factors can influence the profile and content of phenolic compounds, as well as the extraction methods, as discussed in Section 3.1.

Procyanidin B2, epicatechin, and catechin, together, accounted for 93.67% of the total phenolic compounds quantified, reinforcing the remarkable predominance of flavan-3-ols in the araticum pulp. Therefore, the phenolic composition of araticum pulp is strongly characterized by the presence of flavonoids, especially epicatechin and procyanidin B2, which constitute the subclass flavanols and may contribute significantly to its bioactive potential.

### 3.5 Antioxidant Capacity

ABTS^•+^, DPPH, FRAP, and ORAC assays are rapid methods based on different reaction mechanisms and are often used to determine antioxidant capacity.^54^ Table 5 shows that T-ORAC exhibited remarkable activity (551.17 μmol TE/g dw), followed by comparable activities for FRAP, ABTS^•+^, and DPPH (238.46, 257.40, and 229.23 μmol TE/g dw, respectively). These results indicate that the phenolic compounds in araticum pulp exhibit stronger antioxidant capacity through hydrogen atom transfer mechanisms, as assessed by ORAC, compared with single-electron transfer mechanisms (DPPH, ABTS^•+^, and FRAP).

**Table 5:** Antioxidant Capacity of the Araticum Pulp^a^

**Assays**	**Araticum pulp**
DPPH (μmol TE/g dw)	229.23 ± 4.94
ABTS^•+^ (μmol TE/g dw)	238.46 ± 5.40
FRAP (μmol TE/g dw)	257.40 ± 17.36
H-ORAC (μmol TE/g dw)	320.28 ± 4.88
L-ORAC (μmol TE/g dw)	230.88 ± 28.24
T-ORAC (μmol TE/g dw)	551.17 ± 29.51
^•^OH (IC_50_ μg/mL dw)	0.27 ± 0.02
HOCl (IC_50_ μg/mL dw)	99.30 ± 6.26
^•^O_2_^®^ (IC_50_ μg/mL dw)	457.21 ± 0.61

a ABTS: 2,2′-azino-bis (3-ethylbenzothiazoline-6-sulfonic acid); dw: dry weight; DPPH: 2,2-diphenyl-1-picrylhydrazil; FRAP: ferric reducing antioxidant power; HOCl: hypochlorous acid scavenging activity; IC_50_: extract concentration that resulted in a 50% reduction in radical concentration compared to the control; ORAC: oxygen radical absorbance capacity; H-ORAC: hydrophilic ORAC; L-ORAC: lipophilic ORAC (calculated as the difference between T-ORAC and H-ORAC); T-ORAC: total ORAC; ^•^O_2_^®^: superoxide radical scavenging activity; ^•^OH: hydroxyl radical scavenging activity; and TE: Trolox equivalents.

Similar trends have been reported in previous studies. For instance, Arruda et al.^32^ observed higher T-ORAC values (1593.72 μmol TE/g dw) compared with ABTS and DPPH (683.65 and 609.58 μmol TE/g dw, respectively). Likewise, Arruda, Pereira, de Morais et al.^17^ reported H-ORAC, ABTS^•+^, and DPPH values of 259.26, 214.38, and 132.73 μmol TE/g dw, respectively. More recently, Arruda et al.^49^ found H-ORAC and ABTS^•+^ values of 122.29 and 91.02 μmol TE/g fw, respectively.

The hydrophilic fraction (H-ORAC) demonstrated greater peroxyl radical (ROO^•^) scavenging capacity (320.28 μmol TE/g dw) compared to the lipophilic fraction (L-ORAC; 230.88 μmol TE/g dw). Arruda, Pereira, and Pastore^25^ reported a similar H-ORAC value (337.25 μmol TE/g dw), but higher L-ORAC and T-ORAC values (565.02 and 902.27 μmol TE/g dw, respectively). The antioxidant action of the lipophilic fraction may be attributed, at least in part, to the carotenoid content in araticum pulp, particularly moderate levels of β-carotene and lutein (see Section 3.2). Carotenoids are effective scavengers of radicals such as peroxyl, as the conjugated double bonds in their structure can accommodate unpaired electrons.^55^

The results presented in Table 5 also demonstrate a strong antioxidant effect of araticum pulp against ^•^OH, as indicated by the very low IC_50_ value (0.27 μg/mL dw). This radical is one of the most potent oxidants due to its molecular instability, short half-life, and high affinity for biomolecules, which results in immediate cellular damage,^56^ highlighting the importance of araticum pulp as an effective scavenger of ^•^OH.

Additionally, araticum pulp exhibited effective scavenging activity against HOCl, followed by a lower but still relevant activity against ^•^O_2_® with IC_50_ values of 99.30 and 457.21 μg/mL dw, respectively. HOCl plays a key role in host defense against microorganisms, although in excess it can lead to tissue damage, whereas ^•^O_2_® can initiate a cascade of reactions that lead to the formation of secondary ROS, such as hydrogen peroxide (H_2_O_2_), hydroxyl radical (^•^OH), and singlet oxygen (^1^O_2_).^35^

ROS and reactive nitrogen species (RNS) are naturally generated byproducts of aerobic metabolism and play essential roles in cellular signaling, redox homeostasis, and the regulation of transcription factors.^55^ Nevertheless, their excessive production can trigger oxidative stress, resulting in structural and functional damage to biomolecules (lipids, nucleic acids, and proteins), cells, and tissues, as well as the activation of intracellular pro-inflammatory signaling pathways, such as NF-κB and MAPKs.^2,57^ This process, in turn, further increases the production of ROS and RNS, establishing a vicious cycle of stress that amplifies inflammatory responses and contributes to tissue destruction and the development and progression of several chronic diseases, including inflammation, diabetes, cancer, cardiovascular disorders, and neurodegenerative diseases.^57,58^

In this context, phenolic compounds act as key natural antioxidants, protecting cells against the harmful effects of ROS and RNS through hydrogen atom and/or single electron transfer mechanisms, metal ion chelation, regeneration of other antioxidants, and regulation of enzymatic activities.^9,34^ Collectively, these actions prevent and repair DNA damage and modulate signal transduction pathways and gene expression associated with metabolism, proliferation, inflammation, and cell growth, thereby contributing to the reduction of oxidative stress.^58,59^

The present study demonstrated that araticum pulp exhibits remarkable antioxidant capacity, particularly against peroxyl and hydroxyl radicals. This effect is likely attributable to the high abundance and diversity of phenolic compounds in the pulp, which together suggest its potential to mitigate oxidative stress and inhibit inflammatory processes.

### 3.6 Cellular Assessment of Inflammatory Targets

Cytotoxicity assays were performed to determine the araticum pulp extract concentrations for subsequent experiments without compromising cell viability. As seen in Figure 1A, macrophages pretreated with araticum pulp extract did not present reduced cell viability at concentrations up to 1000 μg/mL when compared to the control group (M) (*p* > 0.05), indicating that araticum can be used at relatively high concentrations without causing harmful effects on macrophages.

**Figure 1:**
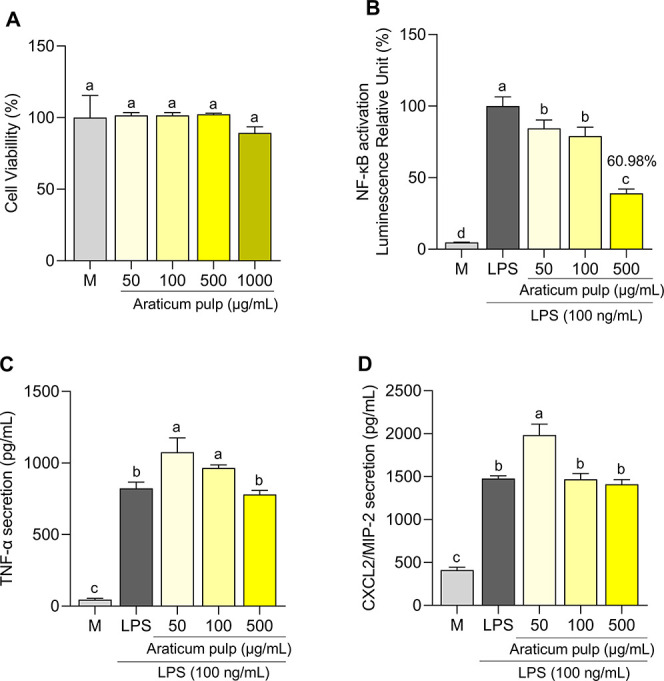
Effect of araticum pulp on cell viability (MTT assay) (A); NF-κB activation (B); and release of TNF-α (C) and CXCL2/MIP-2 (D) in RAW 264.7 cells induced by lipopolysaccharide (LPS; 100 ng/mL). The LPS is a positive control, and M (treated with culture medium) is a negative control. The results were expressed as mean ± standard deviation (*n* = 3); different letters indicate statistical differences (*p* < 0.05), determined by the one-way ANOVA followed by the Tukey test.

NF-κB is a crucial transcription regulator in inflammatory signaling, activated by stimuli such as pathogen-associated molecules, cytokines, enzymes, and oxidative stress. Upon inflammatory stimulation, Toll-like receptor 4 (TLR4) activates IκB kinase (IKK) complex, triggering phosphorylation of inhibitor κBα (IκBα), promoting NF-κB (p65/p50) translocation to the nucleus, where it induces genes encoding pro-inflammatory proteins, like cytokines (e.g., TNF-α, IL-1β, IL-6), chemokines (e.g., CXCL1/KC, CXCL2/MIP-2, and MCP-1), enzymes (e.g., cyclooxygenase-2, and inducible NO synthase), prostaglandins (PGs), and other factors involved in the inflammatory cascade. This coordinated activation amplifies the inflammatory response, while persistent NF-κB dysregulation is strongly associated with chronic inflammatory diseases, including cancer and autoimmune diseases.^7,60^

As shown in Figure 1B, macrophages pretreated with 500 μg/mL of araticum pulp significantly reduced NF-κB activation, resulting in a 60.98% suppression compared with the LPS control group (*p* < 0.05). Epicatechin and procyanidin B2, identified as the major phenolics in araticum, may be involved in this regulation. According to Alsaab et al.^61^ procyanidin B2 suppresses the generation of oxidative mediators by blocking the reduction of phosphorylation of NF-κB p65, thereby preventing its nuclear translocation. Similarly, epicatechin has been shown to downregulate NF-κB and TNF-α expression, attenuating chronic inflammation.^62^

Although the araticum significantly inhibited NF-κB activation, no significant reduction in TNF-α and CXCL2/MIP-2 levels was observed (*p* > 0.05; Figure 1C and D). Using the same cellular model, similar findings have been reported for other phenolic-rich matrices. For instance, bee pollen extract, rich in flavonoids, reduced NF-κB activation without affecting TNF-α or CXCL2/MIP-2 levels.^28^ Likewise, extract from inajá (*Maximiliana maripa*) byproduct, characterized by high levels of procyanidin dimers, catechin, and epicatechin, as well as açaí (*Euterpe oleracea* Mart.) seed extract containing catechin, epicatechin, and procyanidins B1 and B2, were able to reduce NF-κB activation without modulating TNF-α production.^63,64^

On the other hand, Lazarini et al.^65^ reported that the extract from pitangatuba (*Eugenia selloi* B.D. Jacks.), a Brazilian native fruit rich in hydroxybenzoic acids, ellagitannins, and flavonoids, reduced both NF-κB activation and TNF-α levels, but did not affect CXCL2/MIP-2. These findings collectively suggest that phenolic-rich extracts may differentially modulate inflammatory mediators depending on their specific chemical profiles and relative abundance of compounds.

This apparent discrepancy in cytokine modulation may be attributed to compensatory mechanisms and the involvement of alternative signaling pathways, such as MAPKs and JAK-STAT, which also regulate the expression of these cytokines, as seen in Figure 2. In addition, canonical and noncanonical NF-κB pathways and multiple regulatory layers may maintain cytokine production even when NF-κB activation is partially suppressed.^66,67^

**Figure 2:**
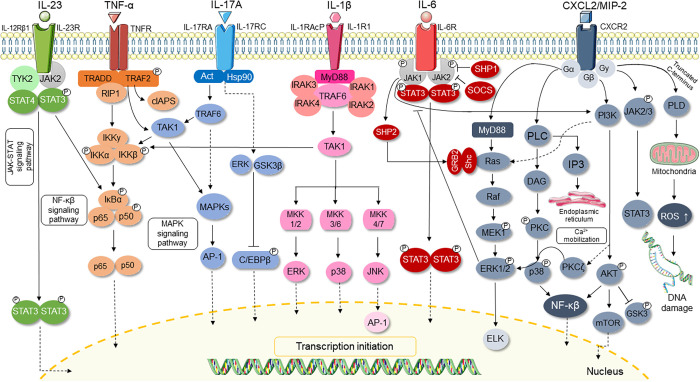
Cytokines, chemokines, and intracellular signaling pathways involved in the activation of inflammation. This is a simplified representation, since there are more connections between the different pathways due to the complexity of the inflammatory response.

The araticum pulp exhibits a complex composition, comprising not only phenolic compounds but also organic acids, jasmonates, iridoids, alkaloids, and sugars, among other constituents.^10^ This chemical heterogeneity may contribute to the inhibition of specific pathways while maintaining others. Furthermore, it is possible that higher concentrations of araticum pulp, beyond those evaluated in the present study, could lead to a reduction in TNF-α and CXCL2/MIP-2 levels. Therefore, further studies exploring a wider concentration range are necessary to better characterize the inhibitory potential of araticum.

The anti-inflammatory activity is largely attributed to the ability of phenolic compounds to inhibit ROS production and modulate cellular oxidative stress.^54^ Arruda et al.^57^ reported that there is a strong correlation between the antioxidant capacity and anti-inflammatory potential of native Brazilian fruits. In this context, the high ROS-scavenging capacity (see Section 3.5), together with the significant reduction in NF-κB activation induced by araticum pulp, indicates that its bioactive compounds can modulate key signaling pathways involved in inflammatory responses. These findings underscore the promising nutraceutical potential of araticum pulp for preventing and managing inflammation-associated disorders.

### 3.7 Molecular Docking Analysis

Docking analysis was performed with the major flavonoids quantified by HPLC-DAD (epicatechin, procyanidin B2, catechin, and rutin) with 13 target proteins, including NF-κB, TNF-α, and CXCL2/MIP-2, and other inflammation-related receptors reported in the literature: IL-6, IL-6R/IL-6, STAT3, IL-1β, IL-1R/IL-1β, IL-23, IL-17A, JAK1, JAK2, and JAK3. Table S3 presents the number of runs, free binding energy (FBE), inhibition constant (*K*_i_), ligand efficiency (LE), number of hydrogen bonds (N° H), bond distances (BD), and involved amino acids in interaction (AAs) of catechin, epicatechin, procyanidin B2, and rutin with the target proteins. Molecular docking results evidenced that, except for procyanidin B2 with IL-6R/IL-6 and JAK1, all evaluated compounds were able to bind to the target proteins (Figure 3).

**Figure 3:**
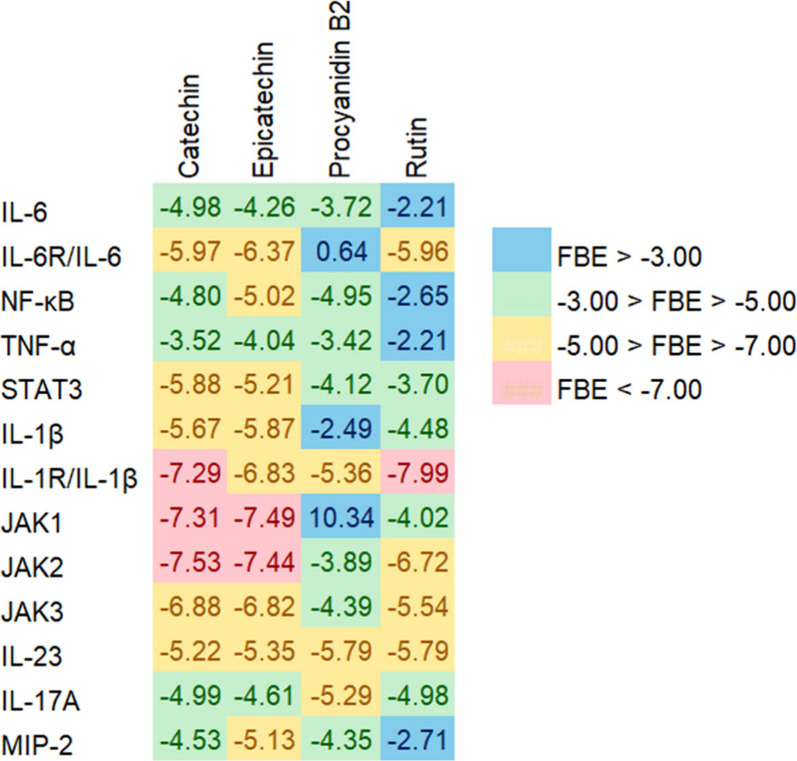
Free binding energy (FBE) heatmap between the main flavonoids from araticum pulp and protein targets involved in inflammatory processes.

Free binding energies below −5.0 kcal/mol are generally considered the threshold for stable ligand–receptor interactions, with lower values indicating greater structural stability.^16^ Additionally, lower *K*_i_ values indicate a stronger inhibitory potency of the ligand with the target protein. Based on this criterion, catechin and rutin exhibited strong binding affinities with IL-1R/IL-1β (−7.29 and −7.99 kcal/mol; *K*_i_ = 4.51 and 1.39 μM, respectively), while catechin and epicatechin showed favorable interactions with JAK1 (−7.31 and −7.49 kcal/mol; *K*_i_ = 4.36 and 3.26 μM, respectively) and JAK2 (−7.53 and −7.44 kcal/mol; *K*_i_ = 2.26 and 3.54 μM, respectively) (Table S3 and Figure 3), indicating a high potential for these compounds to inhibiting target proteins involved in the initial activation of inflammatory signaling pathways (Figure 2).

Hydrogen bonds are facilitators of the protein–ligand, whereas hydrophobic interactions increase the binding affinity of the molecules, helping to stabilize their biochemical environment.^68^ The interaction between catechin and IL-1R/IL-1β involved hydrogen bonds with residues Asp239, Glu105, Lys110, and Met148 (Figure 4A). In turn, rutin and IL-1R/IL-1β forming hydrogen bonds with Glu105, Met148, Asn108, Arg271, Asn204, Gln236, and Thr207 (Figure 4B).

**Figure 4:**
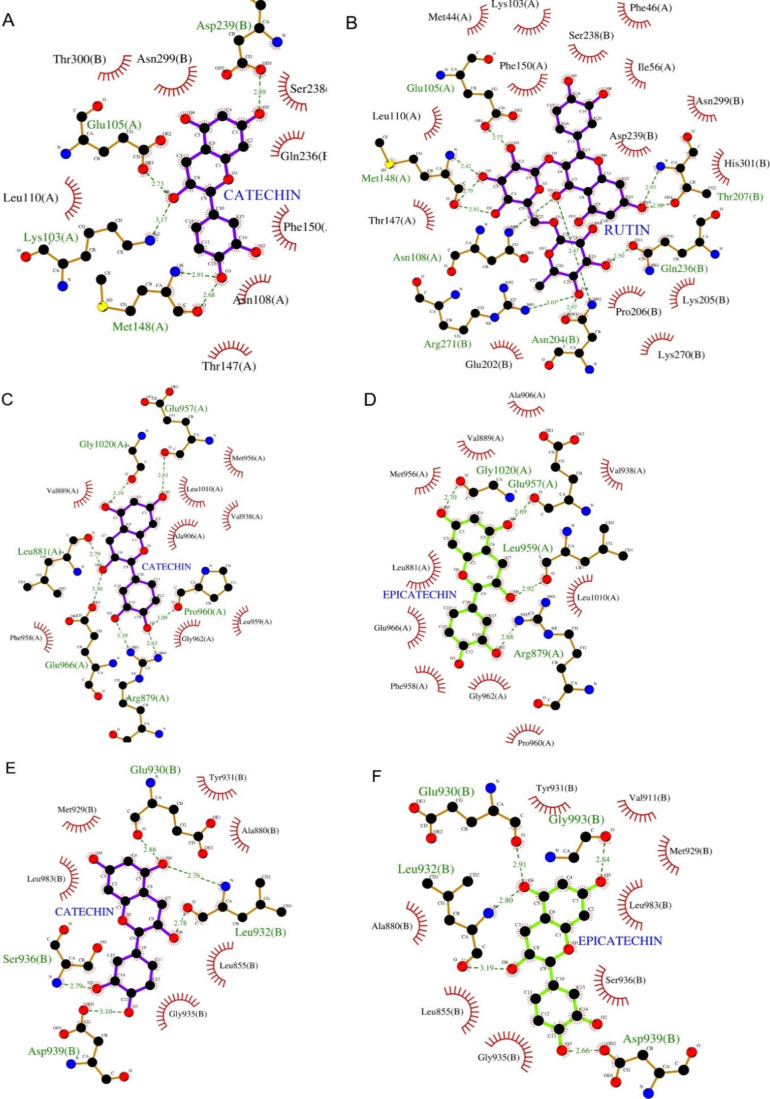
2D representations of the best docking poses showing interactions of catechin, epicatechin, and rutin with target proteins IL-1R/IL-1β (A–B), JAK1 (C–D), and JAK2 (E–F). Catechin and rutin are shown in purple, epicatechin in green, hydrogen bond-interacting amino acids in orange, and amino acids involved in hydrophobic interactions in red.

For JAK1, catechin formed hydrogen bonds with Glu957, Gly1020, Leu881, Glu966, Arg879, and Pro960 (Figure 4C), while epicatechin interacted primarily with Glu957, Gly1020, Leu959, and Arg879 (Figure 4D), indicating an interaction pattern in this target. Similarly, for JAK2, catechin and epicatechin formed hydrogen bonds with Glu930, Leu932, and Asp939 (Figure 4E and F), with additional residues of Ser936 (catechin) and Gly993 (epicatechin), suggesting analogous interaction mechanisms in both isomers. Figure 4 also details the hydrophobic interactions between flavonoids and target proteins, which contributed to the affinity of the complexes.

Epicatechin and catechin were identified as the most versatile ligands, interacting stably with ten and eight proteins, respectively, likely due to their lower molecular weight, which may enhance binding efficiency. Epicatechin exhibited a stable interaction with NF-κB (FBE = −5.02 kcal/mol; *K*_i_ = 97.62 μM), supporting its potential contribution to the NF-κB inhibition observed *in vitro*.

Additional interactions exhibiting low FBE values (<−5.0 kcal/mol) and *K*_i_ values (<100 μM) for other targets can corroborate this inhibition. For example, epicatechin showed strong affinity with IL-1R/IL-1β, JAK3, IL-6R/IL-6, and IL-1β. Likewise, catechin with JAK3, IL-6R/IL-6, STAT3, and IL-1β. Other relevant interactions were observed for procyanidin B2 with IL-23 and rutin with JAK2, IL-6R/IL-6, IL-23, and JAK3 (Table S3).

These interactions suggest that the observed NF-κB inhibition may result not only from direct binding but also from the modulation of interconnected signaling molecules, as illustrated in Figure 2. NF-κB is directly or indirectly regulated through crosstalk with pathways, such as PI3K/AKT, MAPK, and JAK/STAT, related to the cytokine production and modulation of cellular processes.^60,69^ Consequently, the dysregulation of these pathways exacerbates inflammatory processes, establishing a key mechanistic link to the development of chronic diseases.

Molecular docking is fundamental for screening of information about bioactive compounds and target signaling pathways, reducing the need for extensive *in vitro* and *in vivo* assays, optimizing time and costs.^9^ The identification of inflammatory targets establishes a basis for future studies aimed at modulating signaling pathways and developing therapeutic strategies. In this study, only a subset of compounds and target proteins were evaluated. Other ligands and receptors may also contribute to the modulating effects of araticum pulp.

## 4 Conclusions

Araticum pulp exhibits a diverse phytochemical profile comprising carotenoids, organic acids, phenolic acids, and flavonoids. UHPLC-Q-Orbitrap-MS/MS and HPLC-DAD analyses confirmed a rich phenolic composition, mostly of flavonoids such as epicatechin, catechin, rutin, and procyanidin B2. Remarkable antioxidant capacity was observed in araticum pulp, with a highlight for its strong scavenging activity against hydroxyl and peroxyl radicals. In addition, araticum pulp significantly reduced NF-κB activation in RAW 264.7 cells, although no significant effects were observed on TNF-α and CXCL2/MIP-2 release. Molecular docking supported these findings, showing stable interactions between the major flavonoids found in araticum pulp and inflammation-related target proteins, particularly IL-1R/IL-1β, JAK1, and JAK2, suggesting additional mechanisms beyond NF-κB modulation. Together, these findings evidence the antioxidant properties and support the potential modulatory effects of araticum pulp on inflammatory processes, providing valuable insights into its role in mitigating oxidative stress and inflammation-related chronic diseases. Even though the present results are promising, this study was limited to *in vitro* and *in silico* approaches. Future investigations should elucidate the underlying mechanisms across different inflammatory pathways, assess the bioavailability of its phenolics, and explore possible synergistic interactions among its bioactive constituents. Furthermore, preclinical and clinical studies are required to validate and consolidate its antioxidant and anti-inflammatory effects, thereby supporting potential nutraceutical applications.

## Supplementary Material



## Notes

Complementary molecular docking information (PDF)
