# Supplementary material for: Phenolic Compounds from Araticum Pulp (Annona crassiflora Mart.) Modulate Inflammatory Targets: Insights from in Vitro and in Silico Approaches
Source: ACS Omega. 2026 May 19;11(21):30591–605. doi: 10.1021/acsomega.5c12027 (PMC13234800; doi:10.1021/acsomega.5c12027)
Supplement: Supplementary file 1 [file ao5c12027_si_001.pdf]

Supporting Information

Phenolic compounds from araticum pulp (*Annona crassiflora* Mart.) modulate inflammatory targets: insights from *in vitro* and *in silico* approaches

Amanda Cristina Andrade [a, *], Henrique Silvano Arruda [a], Lívia Mateus Reguengo [a], Ana Sofia Martelli Chaib Saliba [b], Severino Matias de Alencar [b], and Glaucia Maria Pastore [a]

[a] Department of Food Science and Nutrition (DECAN), School of Food Engineering (FEA), University of Campinas (UNICAMP), Campinas, 13083-862, São Paulo, Brazil

[b] Department of Agri-Food Industry, Food and Nutrition, Luiz de Queiroz College of Agriculture (ESALQ), University of São Paulo (USP), Piracicaba, 13418-900, São Paulo, Brazil

***Corresponding author:**

Department of Food Science and Nutrition (DECAN), School of Food Engineering (FEA), University of Campinas (UNICAMP), Campinas, 13083-862, São Paulo, Brazil.

E-mail address: andradenut@gmail.com; a183862@dac.unicamp.br (A.C. Andrade)

ORCID: https://orcid.org/0000-0002-0166-1009

Table S1. PubChem CID, molecular weight, and chemical structure of the main flavonoids from araticum pulp.

Compounds	PubChem CID	Molecular Weight	Structure
Catechin	9064	290.27	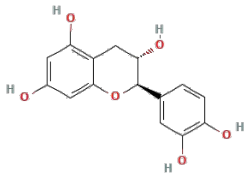
Epicatechin	72276	290.27	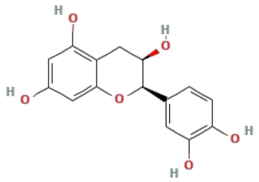
Procyanidin B2	122738	578.50	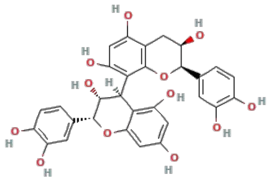
Rutin	5280805	610.50	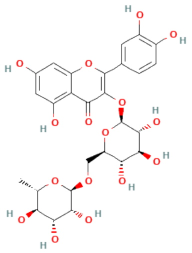

Table S2. Grid box coordinates and size of the target proteins involved in inflammatory processes studied here.

	IL-6	NF-κB	STAT3	TNF-α	IL-1β	JAK1	JAK2	JAK3	IL-23	IL-6R/IL-6	IL-17A	IL-1R/IL1β	MIP2
**Center**													
**X**	0.416	50.354	94.356	-11.865	40.365	13.640	-22.256	-18.867	24.594	-33.639	35.678	48.257	29.328
**Y**	-27.121	117.189	83.804	63.220	9.463	15.088	55.233	34.096	39.121	174.625	-20.012	11.296	-5.641
**Z**	-2.585	54.361	68.609	18.622	57.721	-11.854	-9.880	115.096	37.092	40.246	-0.329	15.893	-0.209
**Size**													
**X**	40	50	50	50	40	40	40	60	60	50	40	50	40
**Y**	40	40	50	40	40	40	40	60	60	50	40	50	40
**Z**	40	50	50	40	40	40	40	60	60	50	40	50	40

Grid box coordinates were calculated using the mean of X, Y, and Z coordinates of each amino acid included in the binding site.

Table S3. Results of docking parameters on the interaction of the main flavonoids from araticum pulp and target proteins involved in inflammatory processes obtained in AutoDock Vina software**.**

	Run	FBE (kcal/mol)	Ki (µM)	LE	N° H	BD	AAs
**IL-6**							
						2.083	HIS164
						1.832	ARG168
Catechin	6	-4.98	224.92	-0.24	5	2.099	GLU51
						2.114	GLU51
						2.115	LYS171
						2.107	HIS164
Epicatechin	18	-4.26	758.96	-0.2	4	2.024	GLU51
						1.648	ARG168
						2.088	LYS171
						1.928	ARG168
						1.993	ARG168
Procyanidin B2	21	-3.72	1.8 mM	-0.09	5	1.902	LYS171
						2.078	GLU51
						2.242	HIS164
						2.148	ARG168
						2.042	ARG168
						1.712	GLU51
Rutin	47	-2.21	24.04 mM	-0.05	6	2.091	LYS171
						1.847	LYS171
						1.820	SER37
**NF-κB**							
						1.919	ARG33
Catechin	49	-4.8	302.63	-0.23	4	2.144	ARG35
						1.812	ILE118
						1.715	ILE118
						2.022	ARG33
Epicatechin	15	-5.02	97.62	-0.26	6	2.056	ARG35
						2.056	ARG35

						1.849	ILE118
						1.662	ILE118
						2.116	ILE118
						2.112	ARG33
Procyanidin B2	36	-4.95	233.55	-0.12	4	1.812	ARG33
						2.075	ARG35
						2.188	ILE118
						2.099	SER45
						2.085	SER45
						2.066	THR52
Rutin	19	-2.65	11.40 mM	-0.06	6	1.839	LYS56
						2.074	SER51
						2.008	ARG33
**STAT3**							
						2.181	THR641
						2.128	THR714
Catechin	29	-5.88	49.10	-0.28	5	1.720	PRO639
						1.925	GLU638
						2.146	GLN644
						2.113	TYR640
Epicatechin	2	-5.21	151.42	-0.25	4	1.971	THR641
						1.878	THR714
						1.685	PRO639
						1.862	THR714
Procyanidin B2	18	-4.12	953.83	-0.1	4	2.110	THR714
						2.112	GLN644
						1.742	ASN647
						1.897	GLU638
Rutin	9	-3.7	1.71 mM	-0.09	3	1.986	GLU638
						2.100	ASN647
**TNF-α**							
Catechin	7	-3.52	2.65 mM	-0.17	1	1.876	TYR151

Epicatechin	13	-4.04	1.10 mM	-0.19	1	1.990	TYR151
Procyanidin B2	17	-3.42	3.10 mM	-0.05	2	1.853	GLN149
						2.230	GLN149
Rutin	6	-2.21	23.80 mM	-0.05	2	1.875	TYR59
						2.175	TYR151
**IL-1β**							
Catechin	22	-5.67	69.51	-0.27	4	2.158	LYS65
						2.093	GLU64
						2.106	GLU64
						2.179	ASN7
Epicatechin	19	-5.87	49.99	-0.28	4	2.148	LEU62
						2.100	ASN7
						1.917	LYS65
						1.872	GLU64
Procyanidin B2	31	-2.49	14.96 mM	-0.06	2	1.880	SER5
						2.118	GLU64
Rutin	5	-4.48	519.59	-0.1	2	1.903	TYR68
						1.887	ASN7
**IL-23**							
Catechin	43	-5.22	149.65	-0.25	3	2.246	HIS48
						1.827	PHE163
						1.834	PHE163
Epicatechin	19	-5.35	120.54	-0.25	2	1.876	PHE163
						2.211	GLN170
Procyanidin B2	38	-5.79	57.46	-0.14	5	1.986	SER316 (E)
						1.999	PHE163
						2.210	PHE163
						1.714	TRP45
						2.180	TRP45
Rutin	11	-5.79	57.02	-0.13	7	2.047	PHE163
						1.787	ASP231 (E)
						1.967	ASP231 (E)

						1.668	TRP45
						2.007	TRP45
						2.235	TYR315
						1.689	PRO49
**IL-17A**							
CATECHIN	29	-4.99	220.85	-0.24	2	1.918	ALA102 (A)
						1.936	THR71 (B)
EPICATECHIN	50	-4.61	420.67	-0.22	4	2.021	THR71 (A)
						2.127	ALA102 (A)
						1.776	ILE100 (A)
						1.806	THR71 (B)
PROCYANIDIN B2	8	-5.29	132.46	-0.13	6	1.996	ASP107 (A)
						1.774	THR71 (A)
						1.768	THR71 (B)
						1.780	THR71 (B)
						2.182	HIS109
						2.060	ASP107 (B)
RUTIN	34	-4.98	223.14	-0.12	3	1.992	THR71 (B)
						2.119	ALA102 (B)
						1.924	ASP107 (B)
**IL-6R/IL-6**							
Catechin	42	-5.97	42.39	-0.28	4	1.868	PRO65 (B)
						2.236	MET67 (B)
						1.811	GLU163 (C)
						2.200	ASP165 (C)
Epicatechin	39	-6.37	21.59	-0.3	6	1.828	SER176 (B)
						1.929	SER176 (B)
						1.909	ARG179 (B)
						2.052	GLN175 (B)
						2.130	LYS54 (B)
						1.758	GLY280 (C)
Procyanidin B2	46	0.64	-	0.02	2	2.134	MET67 (B)
						1.794	GLU163 (C)

Rutin	48	-5.96	42.75	-0.14	6	2.186	SER176 (B)
						1.841	ARG179 (B)
						1.921	ARG179 (B)
						1.811	LYS66 (B)
						2.210	GLN135 (C)
						2.174	GLU163 (C)
**IL-1R/IL-1β**							
Catechin	5	-7.29	4.51	-0.35	7	2.146	LYS103 (A)
						1.959	MET148 (A)
						1.986	MET148 (A)
						1.880	MET148 (A)
						1.811	GLU105 (A)
						1.946	ASP239 (B)
						1.966	SER238 (B)
Epicatechin	8	-6.83	9.93	-0.33	6	2.118	GLU105 (A)
						2.238	MET148 (A)
						2.159	MET148 (A)
						1.716	THR207 (B)
						2.071	THR207 (B)
						2.116	GLN236 (B)
Procyanidin B2	24	-5.36	117.8	-0.13	6	2.087	LYS109 (A)
						2.013	ASN108 (A)
						1.968	ASN204 (B)
						1.693	ASN204 (B)
						2.034	THR207 (B)
						1.980	HIS301 (B)
Rutin	28	-7.99	1.39	-0.19	9	1.582	MET148 (A)
						1.881	MET148 (A)
						2.238	MET148 (A)
						2.111	ASN108 (A)
						1.814	GLU105 (A)
						2.066	THR207 (B)
						2.078	ARG271 (B)

						2.035	GLN236 (B)
						2.064	ASN204 (B)
**JAK1**							
Catechin	18	-7.31	4.36	-0.35	5	1.622/2.176	ARG879
						2.178	PRO960
						1.825	GLU957
						1.875	LEU881
Epicatechin	11	-7.49	3.26	-0.36	5	2.066	PRO960
						1.926	GLU957
						1.880	GLY1020
						2.118	LEU959
						1.883	ARG879
Procyanidin B2	3	10.34	-	0.25	4	1.949	PRO960
						1.806/1.943	ARG879
						0.806	GLU957
Rutin	45	-4.02	343.27	-0.09	6	1.595/1.669	ARG1007
						1.719/2.061	GLU957
						1.734	GLU966
						2.214	PRO960
**JAK2**							
Catechin	34	-7.53	2.26	-0.37	7	1.948/2.146	ASP939
						2.064	GLU930
						1.842/2.035	LEU932
						1.838	SER936
						2.237	GLY993
Epicatechin	1	-7.44	3.54	-0.35	5	1.806/2.188	ASP939
						1.896	LEU932
						1.940	GLY993
						2.077	SER936
Procyanidin B2	19	-3.89	1.41 mM	-0.09	4	1.726/1.853	LEU932
						1.837	ASP994
						2.106	SER936
Rutin	16	-6.72	11.83	-0.16	6	2.033	LEU932

					1.966/2.208	LYS857
					2.086	LEU855
					2.074	GLU930
					1.984	ASP939
**JAK3**						
Catechin	30	-6.88	9.03	-0.33	6	1.773	PHE968
					1.649/1.728	GLU871
					2.194	LEU905
					2.184	GLU903
					2.023	LYS855
Epicatechin	33	-6.82	10.10	-0.22	4	1.842	TYR931
					1.895	ARG980
					1.883	ARG938
					1.918	LEU855
Procyanidin B2	37	-4.39	601.36	-0.1	3	1.726	ASP949
					2.026	ASN832
					1.580	ASP967
Rutin	39	-5.54	87.18	-0.13	7	1.925/1.936	LYS855
					2.126	ASP949
					1.818	ARG953
					2.176	ASN832
					1.939	ASP967
					2.023	ASN954
**MIP2**						
Catechin	2	-4.53	475.32	-0.22	3	1.914	GLN37
					2.078	GLN37
					1.917	THR29
Epicatechin	10	-5.13	172.96	-0.24	3	1.969	ARG8
					1.810	PRO30
					1.803	ARG8
Procyanidin B2	5	-4.35	651.33	-0.1	2	1.815	ARG8
					1.946	CYS35
Rutin	1	-2.71	10.29 mM	-0.06	4	2.088	PRO31

1.753	GLU39
1.666	GLU39
1.710	GLN10

AAs: amino acids involved; BD: bond distance; FBE: free binding energy; Ki: inhibition constant; LE: ligand efficiency; N° H: number of bonds involving hydrogen atoms; Run: number of runs.
